# Development and Application of RAA Nucleic Acid Test Strip Assay and Double RAA Gel Electrophoresis Detection Methods for ASFV and CSFV

**DOI:** 10.3389/fmolb.2021.811824

**Published:** 2022-01-31

**Authors:** Keke Wu, Yuanyuan Zhang, Sen Zeng, Xiaodi Liu, Yuwan Li, Xiaowen Li, Wenxian Chen, Zhaoyao Li, Yuwei Qin, Jinding Chen, Shuangqi Fan

**Affiliations:** ^1^ College of Veterinary Medicine, South China Agricultural University, Guangzhou, China; ^2^ Key Laboratory of Zoonosis Prevention and Control of Guangdong Province, Guangzhou, China; ^3^ Guangdong Laboratory for Lingnan Modern Agriculture, Guangzhou, China

**Keywords:** African swine fever virus, classical swine fever virus, recombinase aided amplification, disposable nucleic acid detection strip, rapid detection

## Abstract

African swine fever (ASF) is an acute, severe and hemorrhagic infectious disease caused by African swine fever virus (ASFV) infecting domestic pigs and wild boars. Since the outbreak of the disease in China in 2018, it has brought a great impact on China’s pig industry. Classical swine fever (CSF) is an acute contact infectious disease of pigs caused by classical swine fever virus (CSFV) infection. Clinically, acute CSF usually shows persistent high fever, anorexia, extensive congestion and bleeding of the skin and mucosa, which are similar to ASF. It is of great significance to prevent, control and accurately detect ASF and CSF in pig farms. In this study, Recombinase aided amplification (RAA) technology combined with a nucleic acid test strip (RAA-strip) was established for simple and specific detection of ASFV/CSFV. The sensitivity and preliminary clinical application results showed that the RAA test strip established in this study could detect recombinant plasmids containing ASFV/CSFV gene fragments as low as 10^3^ copies/µL. The minimum detection limits of virus DNA/cDNA were 10 and 12 pg respectively, and there was no cross-reaction with other porcine viruses. The specificity of the method was good. We used 37–42 clinical samples to evaluate the performance of our established method, and the positive concordance rates with conventional PCR were 94.1 and 57.1%, respectively. In addition, ASFV and CSFV double RAA agarose gel electrophoresis detection methods were established. The results showed that the method had good specificity. The detection limit of this method is 10^6^ copies for ASFV p72 gene recombinant plasmid and 10^5^ copies for CSFV NS5B Gene recombinant plasmid. The use of this method for clinical material detection was consistent with the PCR method. In summary, the developed method of RAA-strip assay for ASFV and CSFV realized the visual detection of pathogens, and the developed method of dual RAA agarose gel electrophoresis assay for ASFV and CSFV realized the simultaneous detection of two pathogens in one reaction, with good specificity, high sensitivity and rapid reaction rate, which was expected to be clinically feasible for the differential diagnosis of ASF and CSF provided technical support.

## Introduction

African swine fever (ASF) is an acute, hemorrhagic, and high contact infectious disease of pigs caused by African swine fever virus (ASFV) (Wang et al., 2021). Swine usually show clinical symptoms such as high fever, respiratory failure, diarrhea after ASFV infection, and punctate hemorrhages can be observed in the skin and internal organs after necropsy of some pigs, whereas pregnant sows often experience abortion and stillbirth, etc. (Tu et al., 2021). The clinical onset of ASF is similar to that of acute cases of Classical swine fever (CSF) at the pathoanatomical examination, and it is often misdiagnosed as CSF when it is diagnosed clinically. China occupies an important international position in pork production and consumption. Since the outbreak of the disease in China in 2018, it has rapidly spread to more than 30 provinces and cities, posing a great threat to China’s economic development and food security (Yue et al., 2009; Guinat et al., 2016; Zhang et al., 2017; Fan et al., 2020a; Wang et al., 2020a). ASFV is highly resistant to the outside world and can persist in blood, secretions, and various environmental pollutants for a long time. ASFV has a large genome and intricate immune escape mechanisms that can be well evaded by the host immune cell clearance, so there are no safe and efficient vaccines and therapeutics, and the way to control the spread of this virus is simply to cull infected animals (Chenais et al., 2019). The ASFV genome contains 150–167 open reading frames (ORFs) and can encode more than 100 protein molecules (Xian and Xiao, 2020). The ASFV p72 protein is encoded by the B646L gene and is located on the surface of the viral capsid. P72 protein has a stretch of very conserved hydrophilic region with little difference in its amino acid sequence among different strains. Due to better stability than other structural proteins of ASFV, p72 protein is often used to establish serological and molecular biological assays (Wen et al., 2019).

CSF is an acute contact infectious disease caused by the classical swine fever virus (CSFV), which often presents with symptoms such as increased body temperature, organ hemorrhage, and immunosuppression, and is classified as animal blight type A by OIE (Li et al., 2019a). Since the discovery of CSF, the CSF epidemic has not been completely eradicated but has been continuously circulating, despite the implementation of comprehensive immunization with CSF vaccines (Zhao et al., 2019). The epidemic characteristics change from the original early acute, typical to the current chronic and atypical, and are susceptible to secondary or concurrent infections with other pathogens (Dixon et al., 2013; Karger et al., 2019; Wang et al., 2020b; Wu et al., 2020). Virulent sows can become infected through vertical transmission, and pregnant sows often present with dystocia, stillbirth, or output of apparently healthy piglets, but they can present with unremarkable mild symptoms during growth, such as poor growth, occasional tremors with weakness or heredity, or anorexia, depression, or mild diarrhea, and many survive for months. Surviving pigs have the potential to become virulent, detoxify long-term, and reinfect gilts causing a vicious cycle (Zhou, 2019). Nowadays, the general prevalence of atypical pig plague causes severe difficulties in the clinical diagnosis of pig plague in China. Therefore, it is important to establish a rapid and efficient detection method for CSF prevention and control and to promote animal husbandry development.

**FIGURE 1:**
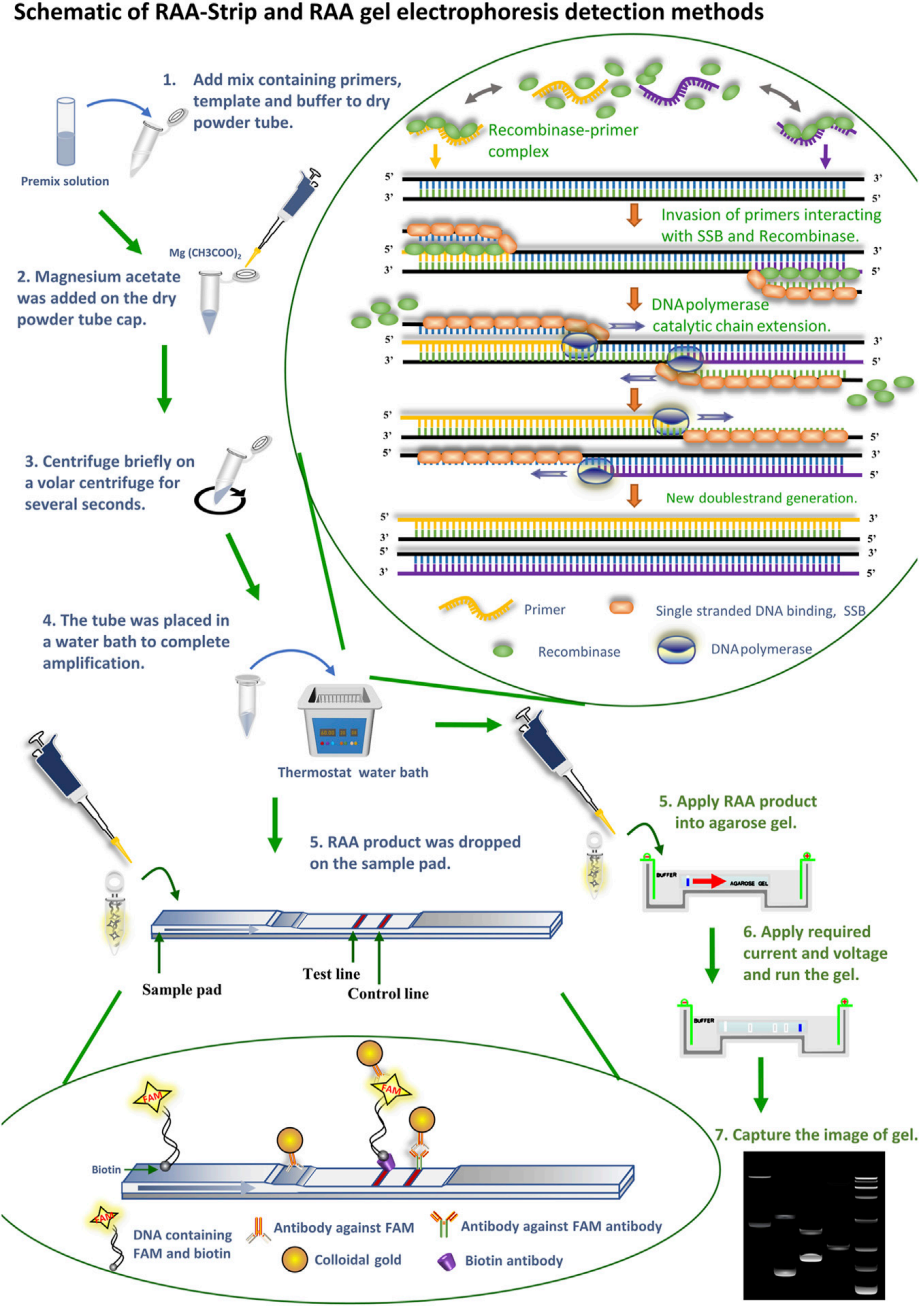
Schematic of RAA-Strip and RAA gel electrophoresis detection methods. Flow of RAA-strip and RAA gel electrophoresis assays are illustrated in the figures in blue and green text, respectively. The reaction principle of the RAA technique is shown in the figure, the recombinase combines with a primer to form a binary complex when the primer corresponds one-to-one to the template DNA at 37–42°C, With the help of single-stranded DNA-binding protein (SSB), the template DNA unwinding becomes two single strands after being separated, and the action of DNA polymerase makes it bind again, at which point a new reaction strand can be synthesized and finally achieving a large amplicons. The primers used above were added with probes to produce FAM and biotin labeled amplicons. Afterwards, DNA molecules containing FAM and biotin dual label were specifically bound to an antibody against FAM labeled with gold particles to constitute a ternary complex, and the complex moved forward continuously under capillary force as the reaction sample mixture moved. When the complex reached the detection line, it was seized by a biotin antibody sprayed on the detection line, at which time the colloidal gold aggregated exponentially and became red. The remaining complex continues to diffuse forward with the solution, and when it reaches the quality control line, FAM antibody sprayed on the quality control line captures it and turns it red.

In recent years, with the updated iteration of molecular biology technology, researchers have established a variety of pathogenic diagnostic methods for ASFV/CSFV (Wen et al., 2019; Choe et al., 2020; Xie et al., 2020; Fan et al., 2020b; Pei et al., 2016; Pei et al., 2013). As standard and routine testing techniques, polymerase chain reaction (PCR), and quantitative PCR (qPCR) techniques are widely used in pathogen inspection and quarantine sites, schools or scientific research institutes, and so on. PCR is a common method for detecting CSF, which can complete the amplification of the fragment of interest using a PCR amplicon under suitable temperature cycles, and some scholars have established a multiplex PCR method that can simultaneously detect six kinds of porcine diseases, and this method provides effective technical support for the detection of mixed infections of CSF with other outbreaks (Yoo et al., 2018). qPCR is a detection technique that uses non-specific SYBR Green I fluorescent dye and specific hydrolyzed fluorescent probe TaqMan to accomplish the quantification of nucleic acids of interest, some scholars have established qPCR assays for the ASFV p72 gene, and the primer-probe sequences involved in the method have been used as recommended sequences by OIE (Zeng et al., 2014). However, these methods have strict requirements for the supporting reaction instrument, and the reaction process is limited by space, cost, etc., thus hindering the use of this technology in the base layers and remote areas. The loop-mediated isothermal amplification (LAMP) (Fan et al., 2020b; King et al., 2003) developed by Japanese scholar Notomi can complete the amplification of target fragments as cauliflower like DNA repeats with different lengths and shapes at a constant temperature of 65°C. Currently, this method has been used for the detection of porcine parvovirus (PPV) (Li et al., 2019b), ASFV (Wang et al., 2021), swine influenza virus (Bakre et al., 2021; Chen et al., 2021), etc. Nevertheless, this technique needs to employ multiple primers for nucleic acid amplification, and it is relatively tedious to design the primer step, which is not suitable for the detection of large batches of samples. Recombinase aided amplification (RAA) technology is the acquisition of a recombinase from bacteria or fungi that enables the *in vitro* amplification of DNA through the co-action with other enzymes capable of replacing the less accessible phage recombinases in recombinase polymerase amplification (RPA) technology. At 37–42°C, the recombinase combines with the primer to form a binary complex, when the primer has a one-to-one correspondence with the template DNA, which, with the help of the single stranded DNA binding protein (SSB), becomes two single strands after the template DNA unwinds apart, and the action of the DNA polymerase causes it to bind again, at which point a new reaction strand can be synthesized, Substantial amplification of the product is eventually achieved. By adding the probe to the RAA reaction system to generate amplification products labeled with 6-carboxy-fluorescein (FAM) and Biotin, followed by placement of the amplification products on a disposal nucleic acid test strip sample pad, rapid visualization of the color development of the test strips in buffer can be achieved (Figure 1) (Wang et al., 2020c). The enzymes required for the RAA reaction have been all premixed and freeze-dried into a dry powder state placed in a 0.2 ml EP tube, which is more convenient to use than liquid reagents and also reduces experimental material losses due to pipetting errors. Enzymes in the dry powder state are also more convenient in terms of transportation, which effectively avoids the decrease in the efficiency of enzymes caused by repeated freeze thawing during the application process. The core of RAA technology is the design and screening of primers and probes, which requires primer length ranging from 30 to 35 bp and probe length usually ranging from 46 to 52 bp, at which point the amplification reaction can be guaranteed to proceed smoothly. This technique has the advantages of rapid detection, easy operation, high sensitivity, high specificity and accurate results, and is more suitable for use in non-laboratory sites with large sample sizes because it has no complex temperature requirements.

Both ASF and CSF are prevalent porcine diseases in China with similar disease onset characteristics, and after infection of pigs, they all suffer from high fever, anorexia, diarrhea, death, and other symptoms. ASF has ravaged in recent years, and there is no effective vaccine for the prevention and control of ASF. Although mandatory immunization of CSF has been adopted in our country, atypical CSF is still in constant prevalence. Current modalities for detecting these two outbreaks are mostly limited to laboratory tests, which are laborious and have longer reaction times. Therefore, there is a critical need to establish an on-site detection modality for use in production practices.

## Materials and Methods

### The Virus and Plasmid

Viral DNA was extracted using the E.Z.N.A. Viral DNA kit (OMEGA, USA) and stored at −20°C. The genomic DNA or cDNA of Japanese encephalitis virus (JEV, SW/GD/2009 strain), Porcine Parvovirus (PPV, GD strain), CSFV (Shimen strain), Pseudorabies Virus (PRV, AH strain), Porcine circovirus type 2 (PCV2, YHW strain), Senecavirus A (SVA, GD-ZYY02-2018 strain), and Foot and Mouth Disease Virus (FMDV) were deposited by the Department of Microbiology and Immunology, College of veterinary medicine, South China Agricultural University. DNA of ASFV was a gift from other laboratories. ASFV p72 gene (Genbank:MK333180.1) was synthesized and cloned into the pMD19-T vector by Sangon biotech (Shanghai) company limited. The synthetic plasmid was named pMD19-T-ASFV p72. The pMD19-T-CSFV NS5B was also deposited by our laboratory.

The recombinant plasmid pMD19-T-ASFV p72 and pMD19-T-CSFV NS5B were extracted using E.Z.N.A Plasmid Mini kit I (OMEGA, USA). The concentrations of recombinant plasmids were determined using NANODROP 2000 Spectrophotometer (Thermo Scientific, USA), and the recombinant plasmids were diluted from 10^10^ to 10^1^ copies/µL (prepared by 10-fold serial dilution) for subsequent sensitivity analysis. The copy number of recombinant plasmids was calculated according to the formula:

$$numbers\;of\;copies\;\left(copies/\mu L\right)=\frac{6.02\times 10^{23}\times DNA\;concentration\;\left(ng/\mu L\right)}{length\;\left(bp\right)\times 10^{9}\times 660}$$

### ASFV/CSFV Conventional PCR Method

Primers for the detection of ASFV p72/CSFV NS5B genes were designed according to the Handbook of World Organisation for Animal Health (OIE) for terrestrial animal diagnostic tests and vaccines and sent to the Sangon biotech (Shanghai) company limited for synthesis (Supplementary Table S1), with amplified fragment lengths of 278 bp and 449 bp, respectively. The PCR reaction system is described as below: A 25 µL volume of PCR amplification reaction consisted of 22 µL of Golden Star T6 Super PCR Mix (Beijing Tsingke Biotechnology Co., Ltd., China), 1 µL of each of the primers (10 µM), and 1 µL of DNA template. The PCR programs were as follows: 98°C for 2 min, then 30 cycles of 98°C for 10 s, 57°C for 10 s, 72°C for 10 s, and a final extension at 72°C for 1 min. Amplicons were analyzed by electrophoresis using 1% agarose gels.

### ASFV/CSFV qPCR Method

Specific primers for qPCR detection were designed and synthesized for the ASFV p72 gene (GenBank: mk333180.1) and CSFV NS5B gene (GenBank: ay775178.2) (Supplementary Table S2), with amplified target gene lengths of 80 bp and 174 bp, respectively. The qPCR reaction system is described as below: A 20 µL volume of qPCR amplification reaction consisted of 10 µL of ChamQ SYBR qPCR Mix (Vazyme Biotech Co., Ltd., China), 0.4 µL of each of the primers (10 µM), 8.2 µL of ultrapure water and 1 µL of DNA template. The qPCR programs were as follows: Pre-denaturation at 95°C for 30 s, denaturation at 95°C for 10 s, and extension at 60°C for 30 s, with a total of 40 cycles set up.

### Design of RAA Primers and Probe

The primers were designed by Primer 5.0 and Oligo 7 for the conserved regions of ASFV p72 gene, CSFV NS5B gene according to the primer design principles of the RAA nucleic acid amplification kit (Jiangsu Qitian Gene Biotechnology company limited, China), which ensured that the designed primers amplified efficiently and had fewer dimers and hairpin structures between primers. The designed primers of ASFV and CSFV were screened by RAA agarose gel electrophoresis using ASFV p72 plasmid, CSFV NS5B plasmid as template, and ultrapure water as negative control, respectively. The reaction system and reaction procedure were as follows: a mixture containing 25 µL Buffer V, 15.7 µL ultrapure water, 2.4 µL forward primer (10 µM) and 2.4 µL reverse primer (10 µM) were added to a reaction tube containing lyophilized enzyme powder to dissolve the lyophilized powder. Subsequently, 2 µL DNA samples and 2.5 µL magnesium acetate solution (280 mM) were added into the tube. The above reaction tube was placed in a constant temperature water bath at 39°C to react for 15–30 min. After completion of amplification, the product was mixed evenly with an equal ratio of chloroform solution and the mixture was centrifuged (12,000 rpm) at room temperature for 5 min, 1.5% agarose gel electrophoresis was used to detect the amplification products in the supernatant. In addition, based on the design principle of the RAA probe (Kim et al., 2021), probe design was performed for the primers obtained after screening, and special biomarkers were added for the primers and probes. All primers and probes were synthesized by Sangon Biotech (China).

### ASFV/CSFV RAA-Strip

After obtaining the optimal RAA primer and probe, the RAA assay combined with a disposable nucleic acid detection strip (RAA-strip) for ASFV/CSFV was established by using ASFV p72 plasmid and CSFV NS5B plasmid as a template, respectively, and ultrapure water as a negative control. The RAA reaction system was as described in 2.4 (Figure 1), and 10 µL RAA product was dropped into the disposable nucleic acid test strip sample pad. The strips were then placed into a slide containing 100 µL tubes of buffer (Ustar Biotechnologies, China), and the results were judged within 15–30 min. Both the quality control line (C) and the test line (T) with bands were judged as positive, and only the quality control line (C) with bands was judged as negative, otherwise as invalid results.

### Optimization of Reaction Condition for RAA-Strip

To obtain optimal reaction temperature for the RAA assay, the RAA reaction system was placed in a water bath with different temperatures (25–45°C) for 15 min. Then, nucleic acid test strips were used to detect the results. After obtaining the optimal temperature, the RAA assay was performed with different incubation time (5–30 min) using the optimal temperature to verify the optimal incubation time.

### Specificity and Sensitivity of RAA-Strip

The specificity of ASFV/CSFV RAA-strip was evaluated using different swine-associated viruses including PCV2, PPV, JEV, CSFV, PRRSV, PRV, SVA, and ASFV. Each trial was repeated at least 3 times.

Plasmid standards pMD19T-p72 and pMD19T-NS5B for ASFV and CSFV, respectively (10^1^–10^10^ copies/µL. Prepared by 10 fold serial dilution) was used as a template for comparison with the qPCR, the RAA agarose gel electrophoresis, and the conventional PCR; ASFV DNA (1 μg–100 fg, prepared by 10-fold serial dilution) and CSFV cDNA (1.2 µg–120 fg, prepared by 10-fold serial dilution) were used as templates to compare well with those detected by conventional PCR; CSFV Shimen strain with TCID_50_ of 10^4.7^/ml was used as the initial strain concentration and diluted 10-fold. The cDNA extracted from the diluted virus solution with different strain concentrations was used as the template. The reaction products were detected by RAA-strip and conventional PCR to explore the virus titer sensitivity of the CSFV RAA-strip detection method. Each test shall be repeated at least 3 times.

### Test of Clinical Samples

DNA extraction was performed on the ASF suspicious samples, RNA extraction was performed on the CSF suspicious samples, and cDNA was obtained by reverse transcription, which was used as a template for separate detection with the ASFV/CSFV RAA-strip, while conventional PCR was used for comparative analysis.

## Results

### Establishment and Initial Application of ASFV RAA-Strip Assay

#### Establishment of AFSV RAA-Strip and Optimization of the Reaction Condition

**FIGURE 2:**
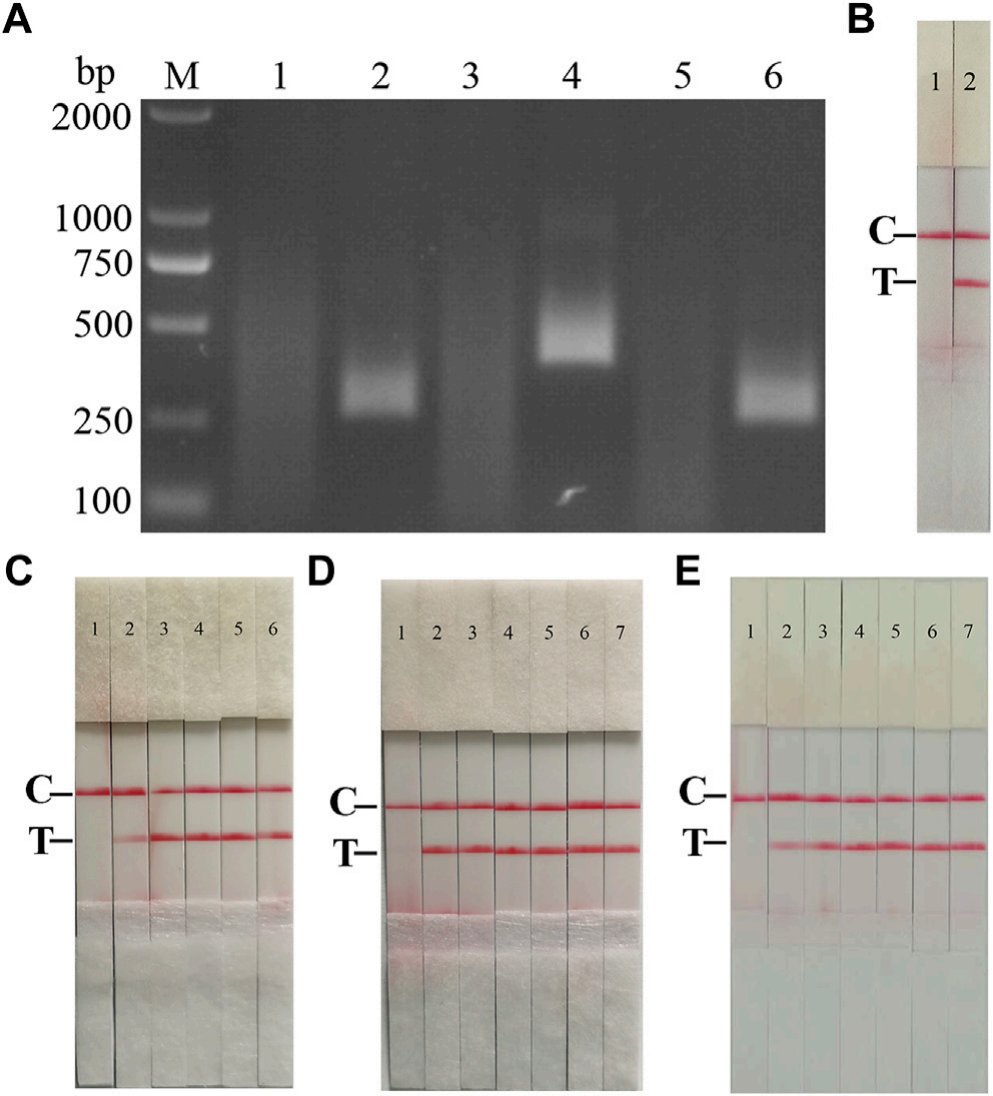
Establishment of AFSV RAA-Strip and Optimization of the Reaction Condition. **(A)** Optimal primer selection. The products amplified by different RAA primers were analyzed by 1.5% agarose gel electrophoresis to select the optimal primer pairs. M is a 2,000 bp DNA marker. The primers and templates used in lane 1–6 successively are: ASFV-RAA-F1/R1+ddH_2_O, ASFV-RAA-F1/R1+ASFV, ASFV-RAA-F2/R2+ddH_2_O, ASFV-RAA-F2/R2+ASFV, ASFV-RAA-F3/R3+ddH_2_O, and ASFV-RAA-F3/R3+ASFV. **(B)** Initial development of the ASFV RAA nucleic acid strip assay. Using the recombinant plasmid pMD19-T-p72 as a template to test the ASFV RAA-strip. C is a control line. T is a test line. The templates used in lane 1and 2 successively are the negative control and the ASFV pMD19-T-p72. **(C)** The determination of the optimal reaction temperature range of ASFV RAA-strip. The temperature of templates used in lane 2–6 successively are 25, 30, 35, 40, and 45°C. **(D)** The detection of the accurate optimal temperature. The temperature of templates used in lane 2–7 successively are 35, 36, 37, 38, 39, and 40°C.**(E)** Optimization of reaction time. Using the recombinant plasmid pMD19-T-p72 as a template to optimize the amplification time of ASFV RAA-strip. Line 1 is a negative control. The reaction time of templates used in lane 2–7 successively are 5, 10, 15, 20, 25 and 30 min.

Following the primer design principles of the RAA nucleic acid amplification kit, applying Primer 5.0 and Oligo 7 against the ASFV p72 gene, three pairs of specific primers for RAA amplification were designed and synthesized (Supplementary Table S3). The RAA reaction was performed according to method 2.2 to detect primer specificity, agarose gel electrophoresis results showed (Figure 2A), three sets of primers showed specific bands corresponding to expectations at around 288, 416, 282 bp, among which the second pair of primers had the brightest band and no heterozygous bands. The second primer pair was therefore identified as the optimal primer for ASFV RAA analysis, and probes were designed based on the targeted amplified fragment. Details regarding the probes and primers are listed in Table 1.

**TABLE 1:** Optimal primer pairs and probe for AFSV recombinase aided amplification (RAA).

Primer name	Sequence (5′-3′)	Position (nt)
ASFV-RAA-F2	CAA​GCC​GCA​CCA​AAG​CAA​ACC​TAT​TCT​TAC​CG	747–778
ASFV-RAA-R2-B	Biotin-AGGTTCACGTTCTCATTAAACCAAAAGCGCA	1028–996
ASFV-Probe	FAM-CCAAAGCAAACCTATTCTTACCGATGAAAA-THF-GATACGCAGCGAACG-C3 spacer	756–801

The RAA reaction was performed with the ASFV p72 plasmid as the template and ultrapure water as the negative control. At the end of the reaction, 10 µL were taken from the amplification products and added to the test strip sample pad, and the test strip was placed in the buffer for 5 min. The results showed (Figure 2B), both C-line and T-line of the pMD19-T-p72 showed red bands, and the negative control only C-line showed red bands, indicating that the RAA nucleic acid dipstick assay method of ASFV was preliminarily established.

In order to determine the optimum reaction temperature of ASFV RAA-strip method, the incubation time was set to 30 min, and RAA amplification reaction was carried out at different temperatures of 25, 30, 35, 40, and 45°C, respectively. It was found that the optimal reaction temperature range of the test was 35–40°C, and 39°C was the optimal reaction temperature of the method (Figures 2C,D). Further, different incubation times of 5, 10, 15, 20, 25, and 30 min were tested at 39°C, and it was found that the red band on the detection line was obvious after 15 min therefore, 15 min was selected as the incubation time of this method (Figure 2E).

#### Specificity and Sensitivity of ASFV RAA-Strip

**FIGURE 3:**
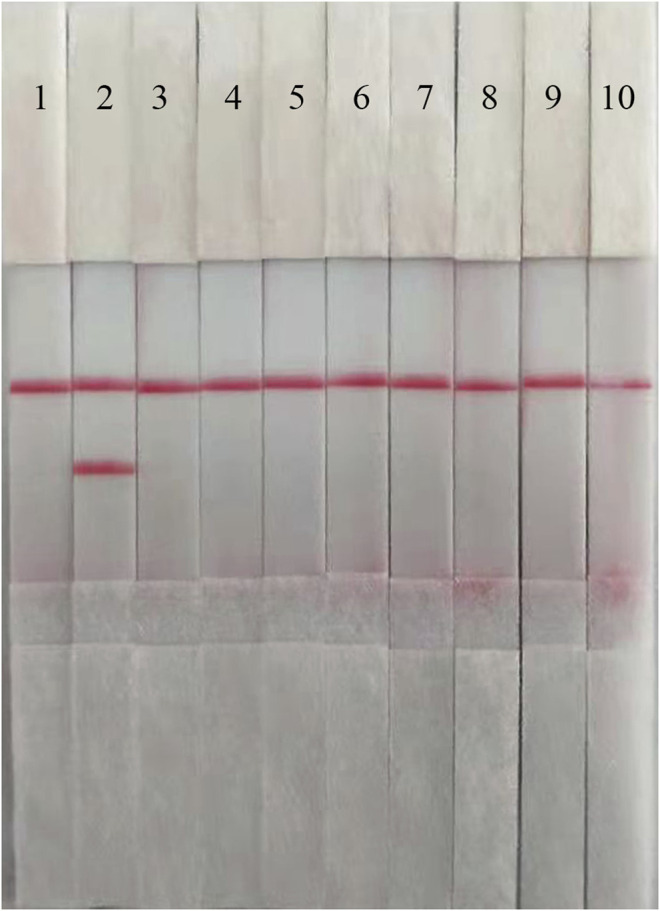
The specificity of ASFV RAA-strip. C is a control line. T is a test line. Lane 1 is a negative control. The specificity test result of ASFV RAA-strip using 9 different pig viruses, including ASFV, CSFV, PPV, (PCV2, FMDV, SVV, PRV, PRRSV, JEV.

To confirm the specificity of the ASFV RAA-Strip, a reaction was performed using ASFV, CSFV, PPV, PCV2, FMDV, SVV, PRV, PRRSV, and JEV as templates, respectively. As shown in Figure 3, the T line appeared for the ASFV-positive sample, and no cross-reaction was observed with other pathogens related to pigs. The results indicated that the specificity of the ASFV RAA-Strip assay was good.

**FIGURE 4:**
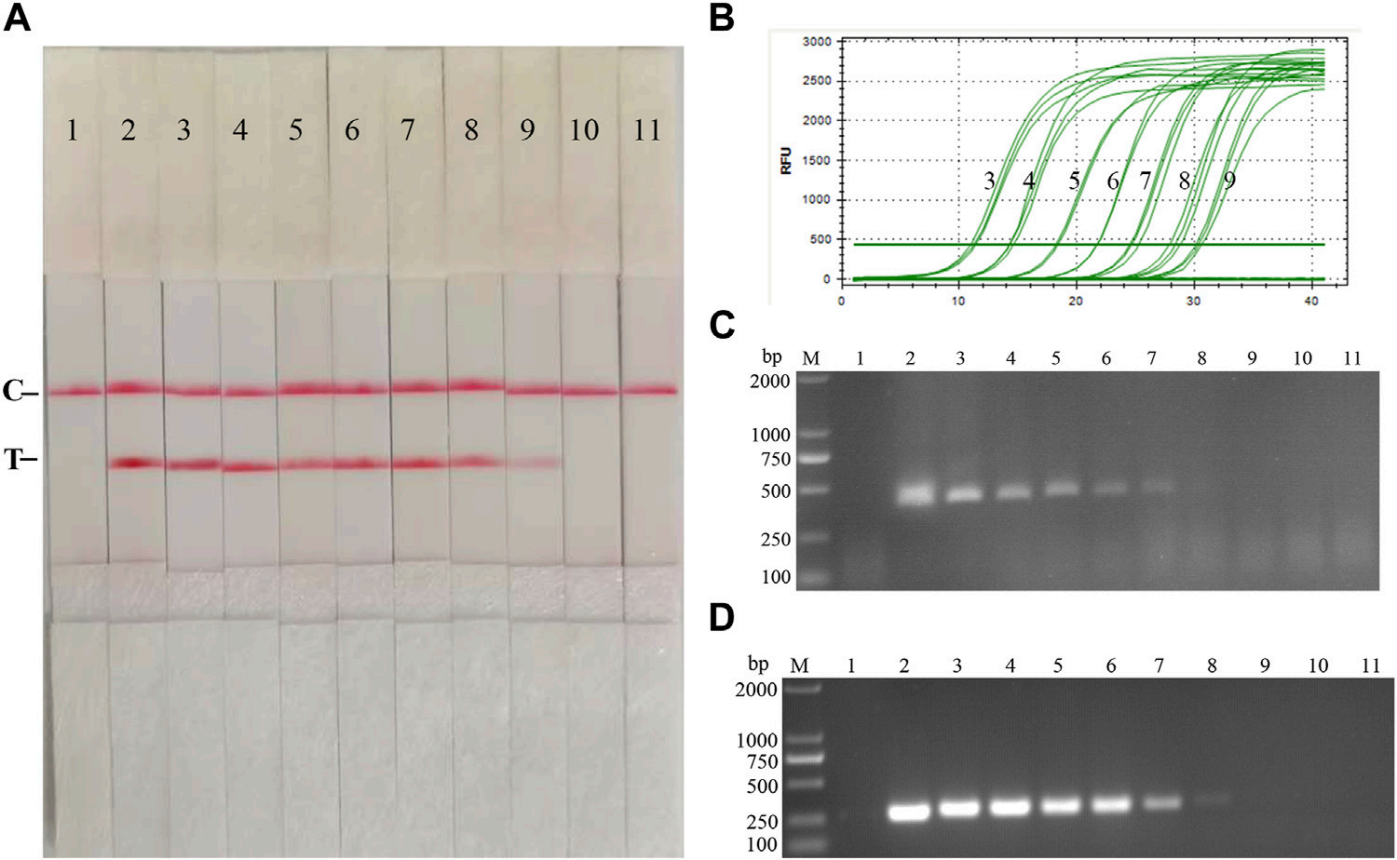
Recombinant plasmids sensitivity analysis of ASFV RAA-strip. **(A)**The sensitivity test result of plasmid copy number by ASFV RAA-strip in order. **(B–D)** are that by the quantitative fluorescent PCR assay, the RAA agarose gel electrophoresis assay, and the conventional PCR assay. C is a control line. T is a test line. M is a 2,000 bp DNA marker. The copies of templates used in lane 1–11 successively are negative control, 10^10^, 10^9^, 10^8^, 10^7^, 10^6^, 10^5^, 10^4^, 10^3^, 10^2^, and 10^1^ copies/μL.

Fold dilutions of recombinant plasmids pMD19-T-p72 were used as templates in the assay using the RAA nucleic acid dipstick assay, which was also compared with the quantitative fluorescent PCR assay, the RAA agarose gel electrophoresis assay, and the conventional PCR assay (Figures 4A–D). The results showed that the ASFV RAA-Strip method had the lowest detection limit of 10^3^ copies/µL for ASFV gene copy number, which was consistent with the sensitivity of the qPCR method, 100 fold more sensitive than the ASFV RAA agarose gel electrophoresis method, and 10 fold more sensitive than the conventional PCR method, indicating that the ASFV RAA nucleic acid test strip assay was better sensitive.

**FIGURE 5:**
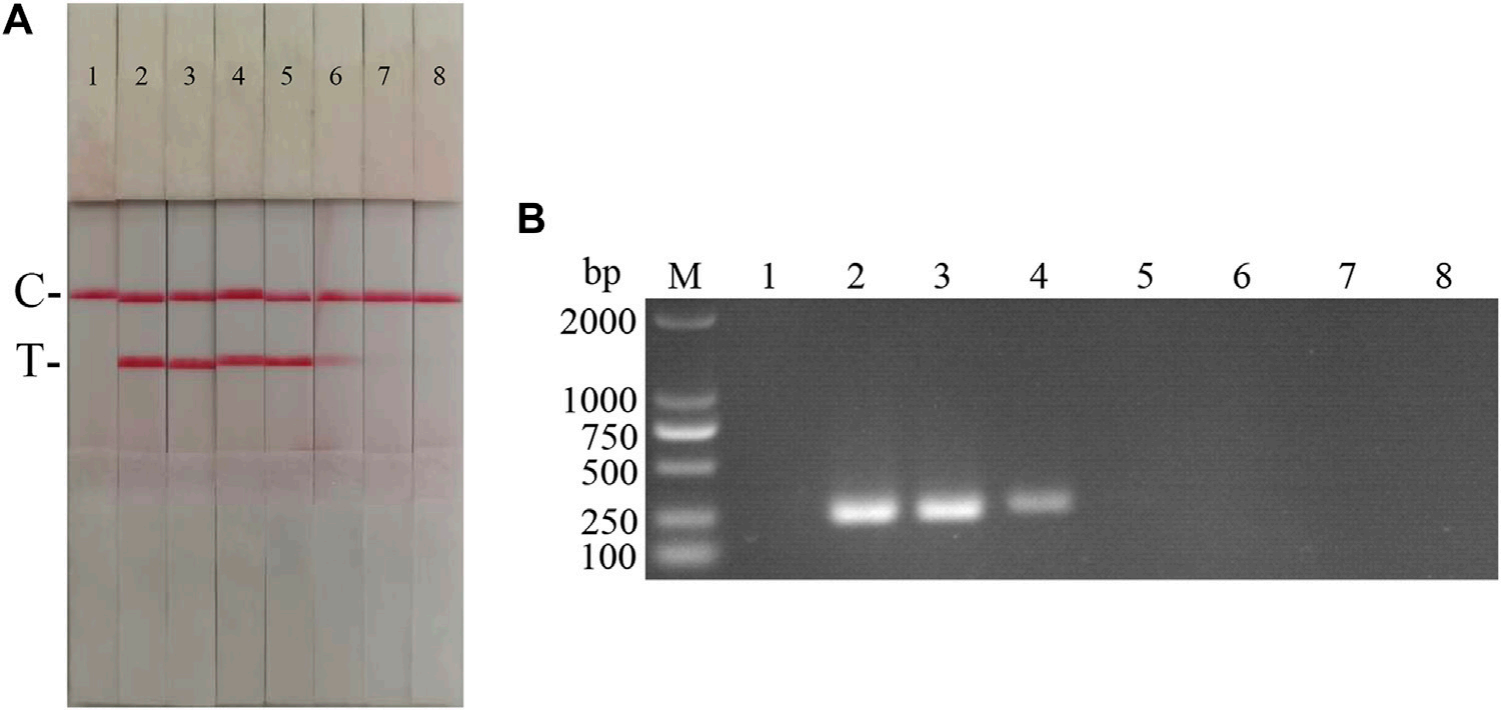
DNA sensitivity analysis of the ASFV RAA-strip assay. **(A)** The sensitivity test result of ASFV DNA by ASFV RAA-strip in order. **(B)** was that by the conventional PCR assay. C is a control line. T is a test line. M is a 2,000 bp DNA marker. The templates used in lane 1–8 successively are: negative control, 100 μg/ml, 10 μg/ml, 1 μg/ml, 1 × 10^−1^ μg/ml, 1 × 10^−2^ μg/ml, 1 × 10^−3^ μg/ml, 1 × 10^−4^ μg/ml.

Fold ratio diluted ASFV DNA was used as the reaction template. The results (Figure 5) showed that the ASFV RAA-strip assay could detect DNA at a minimum concentration of 1 × 10^−2^ μg/ml, that is, the lowest detection limit of DNA content was 10 pg, which was 100 fold more sensitive than the conventional PCR method to detect DNA.

#### Clinical Samples Detection

**FIGURE 6:**
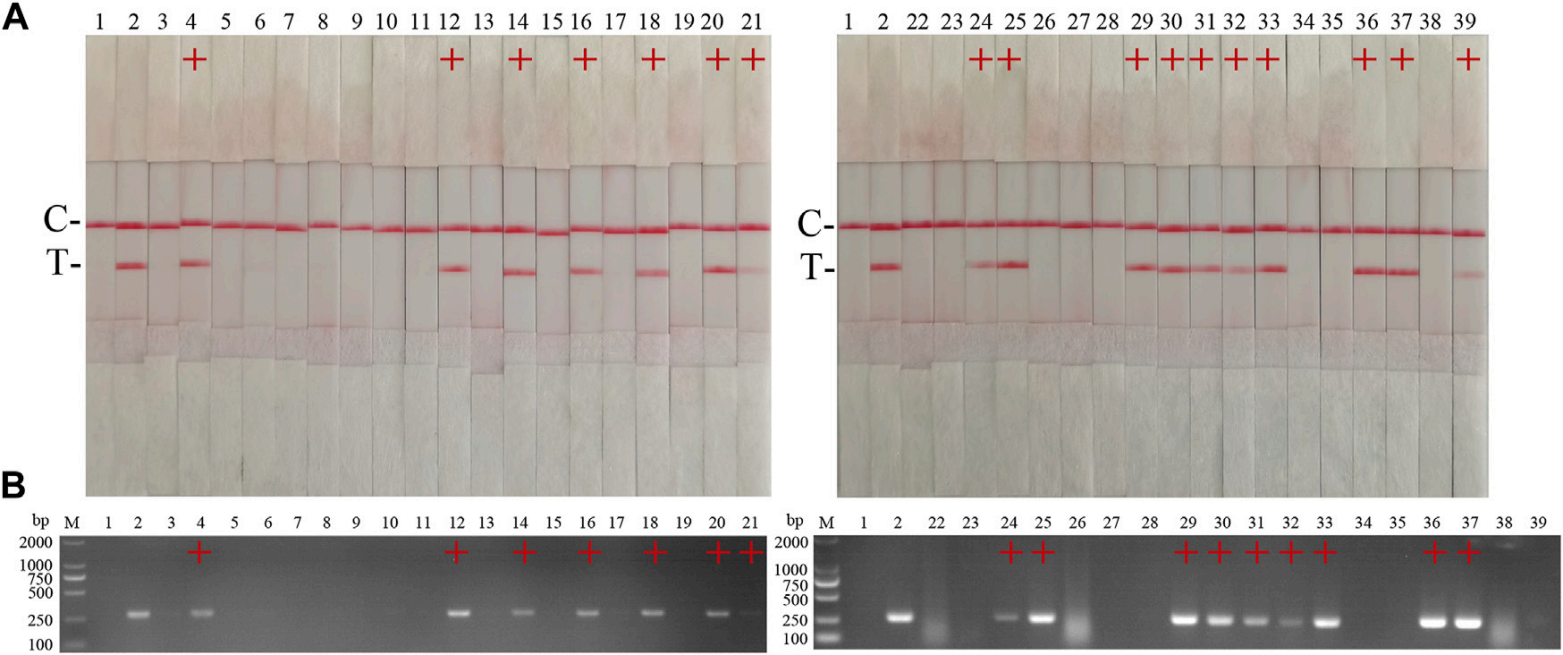
Performance of ASFV RAA-strip on clinical samples. **(A)** The result of clinical samples detection by ASFV RAA-strip. **(B)** The result of clinical samples detection by ASFV conventional PCR. C is a control line. T is a test line. M is a 2,000 bp DNA marker, 1 is a negative control. 2 is positive control (3–39) are different clinical pig samples reserved in our laboratory, and “+” indicates positive detection of ASFV in the samples.

The performance of the ASFV RAA-strip was evaluated using 37 clinical samples and compared with that of conventional PCR. As shown in Figure 6; Table 2, among these samples, 17 positive samples and 20 negative samples were detected by the ASFV RAA-strip method, the positive rate was 45.9% and the negative rate was 54.1%; 16 positive samples and 21 negative samples were detected by conventional PCR. The positive rate was 43.2% and the negative rate was 56.8%. The positive coincidence rate of the two methods was 94.1%, the negative coincidence rate was 95.2%, and the total coincidence rate was 97.3%. It shows that the ASFV RAA-strip detection method has certain clinical feasibility.

**TABLE 2:** Comparison of ASFV RAA-strip and conventional PCR in clinical samples.

Number of samples	ASFV RAA-strip		Conventional PCR		Coincidence rate
	Positive	Negative	Positive	Negative	
37	17	20	16	21	97.3%

### Establishment and Initial Application of CSFV RAA-Strip Assay

#### Establishment of CFSV RAA-Strip and Optimization of the Reaction Condition

**FIGURE 7:**
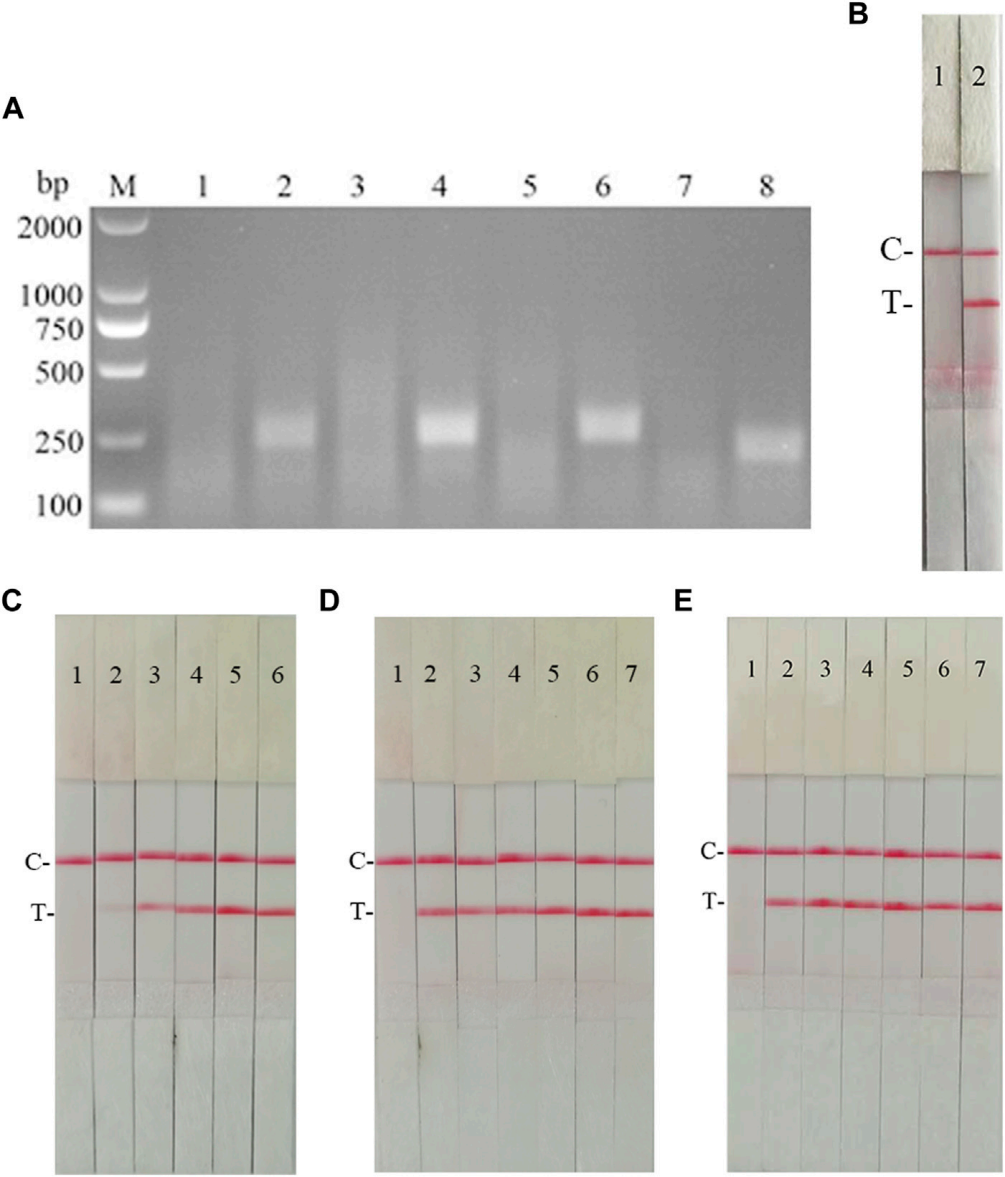
Establishment of CSFV RAA-Strip and Optimization of the Reaction Condition. **(A)** Optimal primer selection. M is a 2,000 bp DNA marker. The primers and templates used in lane 1–8 successively are: CSFV-RAA-F1/R1+ddH_2_O, CSFV-RAA-F1/R1+CSFV, CSFV-RAA-F2/R2+ddH_2_O, CSFV-RAA-F2/R2+CSFV, CSFV-RAA-F3/R3+ddH_2_O, CSFV-RAA-F3/R3+CSFV, CSFV-RAA-F4/R4+ddH_2_O, and CSFV-RAA-F4/R4+CSFV. **(B)** Initial development of the CSFV RAA nucleic acid strip assay. Using the recombinant plasmid pMD19-T-NS5B as a template to test the CSFV RAA-strip. C is a control line. T is a test line. The templates used in lane 1and 2 successively are the negative control and the CSFV pMD19-T-NS5B. **(C)** The determination of the optimal reaction temperature range of CSFV RAA-strip. The temperature of templates used in lane 2–6 successively are 25, 30, 35, 40, and 45°C. **(D)** The detection of the accurate optimal temperature. The temperature of templates used in lane 2–7 successively are 35, 36, 37, 38, 39, and 40°C. **(E)** Optimization of reaction time. Using the recombinant plasmid pMD19-T-NS5B as a template to optimize the amplification time of CSFV RAA-strip. Line 1 is a negative control. The reaction time of templates used in lanes 2–7 successively are 5, 10, 15, 20, 25 and 30 min.

According to the primer design principles of the RAA nucleic acid amplification kit, applying Primer 5.0 and Oligo 7 against the CSFV NS5B gene, four pairs of specific primers for RAA amplification were designed and synthesized (Supplementary Table S4). The RAA reaction was performed according to method 2.2 to detect primer specificity, agarose gel electrophoresis results showed (Figure 7A), four sets of primers showed specific bands corresponding to expectations at around 248, 253, 260, and 207 bp, among which the second pair of primers had the brightest band and no heterozygous bands. The second primer pair was therefore identified as the optimal primer for CSFV RAA analysis, and probes were designed based on the targeted amplified fragment. Details regarding the probes and primers are listed in Table 3.

**TABLE 3:** Optimal primer pairs and probe for AFSV recombinase aided amplification (RAA).

Primer name	Sequence (5′-3′)	Position (nt)
CSFV-RAA-F2	TGA​TGA​TAT​TGA​GTT​TTG​CTC​CCA​TAC​ACC	1831–1860
CSFV-RAA-R2-B	Biotin-TGGTTTCACTTGCAGTTCAGTTGATAGCAC	2083–2054
CSFV- Probe	FAM-ATACACCAATACAAGTAAGGTGGTCAGACA-THF-CACTTCTAGTTACAT-C3 spacer	1854–1899

The RAA reaction was performed with the CSFV NS5B plasmid as the template and ultrapure water as the negative control. At the end of the reaction, 10 µL were taken from the amplification products and added to the test strip sample pad, and the test strip was placed in the buffer for 5 min. The results showed (Figure 7B), both C-line and T-line of the pMD19-T-NS5B showed red bands, and the negative control only C-line showed red bands, indicating that the RAA nucleic acid dipstick assay method of CSFV was preliminarily established.

In order to determine the optimum reaction temperature of CSFV RAA-strip method, the incubation time was set to 30 min, and RAA amplification reaction was carried out at different temperatures of 25, 30, 35, 40, and 45°C, respectively. It was found that the optimal reaction temperature range of the test was 35–40°C and 39°C was the optimal reaction temperature of the method (Figures 7C,D). Further, different incubation times of 5, 10, 15, 20, 25, and 30 min were tested at 39°C, and it was found that the red band on the detection line was obvious after 15 min therefore, 15 min was selected as the incubation time of this method (Figure 7E).

#### Specificity and Sensitivity of CSFV RAA-Strip

**FIGURE 8:**
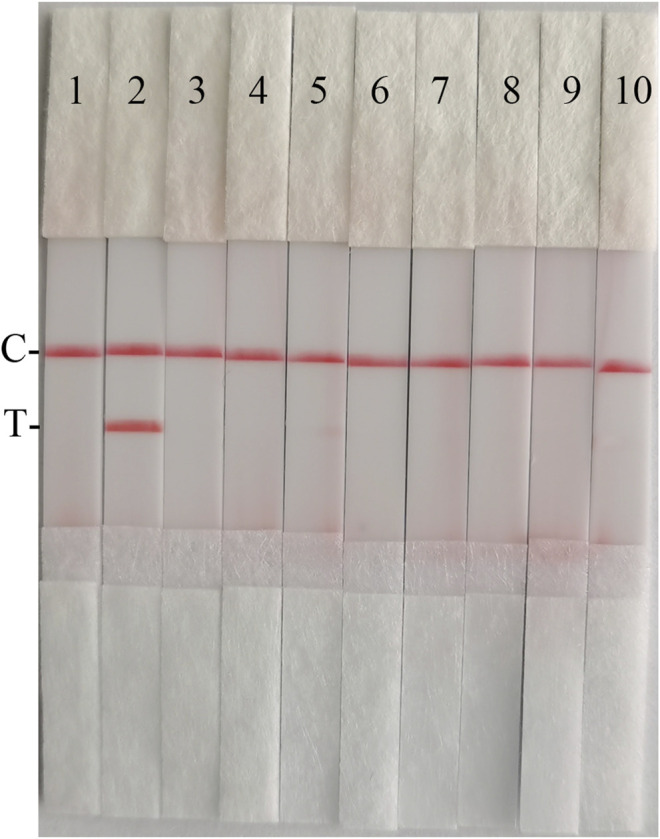
The specificity of CSFV RAA-strip. C is a control line. T is a test line. Line 1 is a negative control. The specificity test result of CSFV RAA-strip using 9 different swine viruses, The templates used in lanes 2–7 successively are CSFV, ASFV, PPV, PCV2, FMDV, SVV, PRV, PRRSV, and JEV.

To confirm the specificity of the CSFV RAA-Strip, a reaction was performed using CSFV, CSFV, PPV, PCV2, FMDV, SVV, PRV, PRRSV, and JEV as templates, respectively. As shown in Figure 8, only CSFV positive templates showed a distinct red band at the T-line, and no specific bands appeared for other pathogens related to pigs. The results indicated that the specificity of the CSFV RAA-Strip assay was good.

**FIGURE 9:**
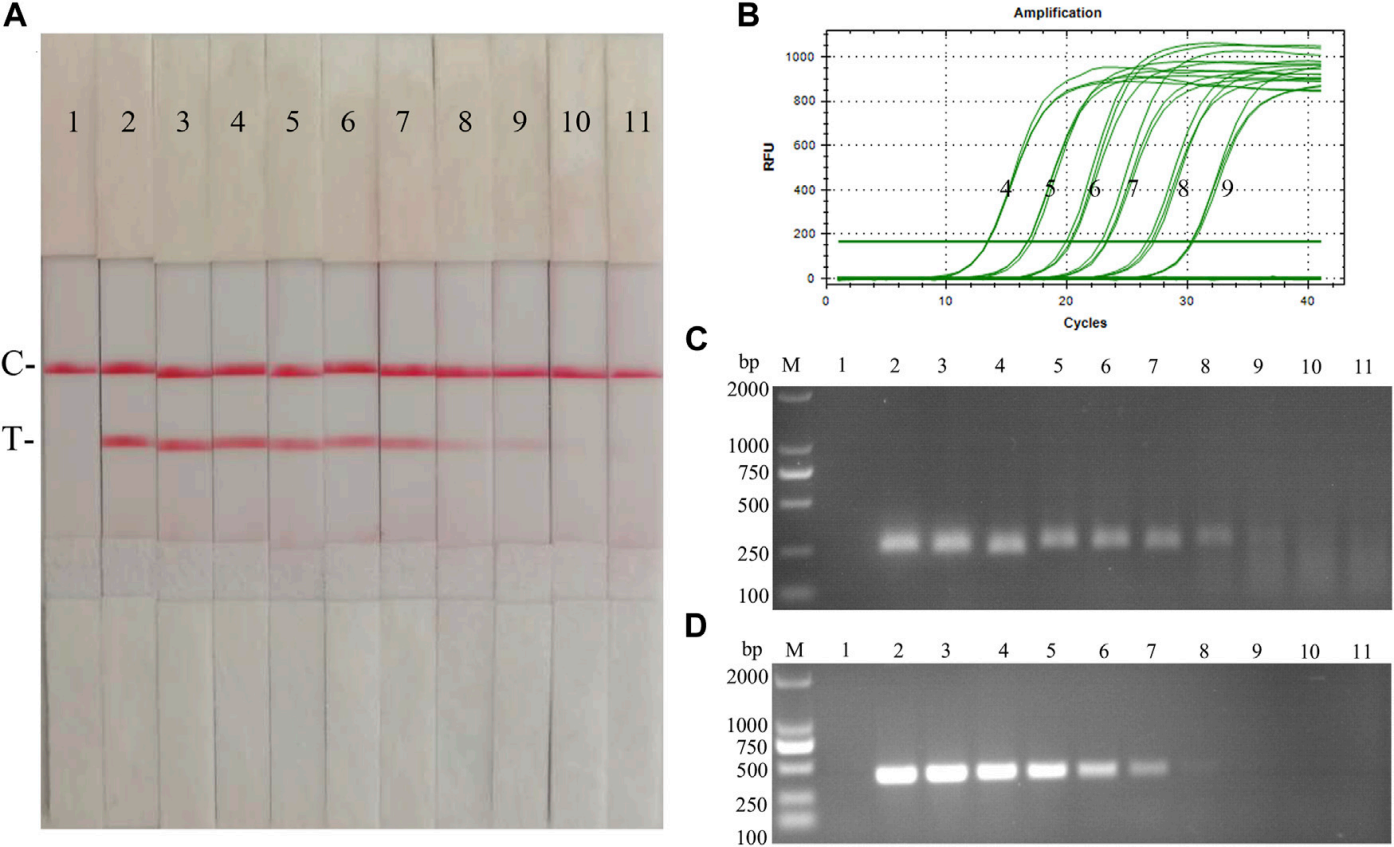
Recombinant plasmids sensitivity analysis of CSFV RAA-strip. **(A)**The sensitivity test result of plasmid copy number by CSFV RAA-strip in order. **(B–D)** are that by the qPCR assay, the RAA agarose gel electrophoresis assay, and the conventional PCR assay. C is a control line. T is a test line. M is a 2,000 bp DNA marker. Line 1 is the negative control, and the copies of templates used in lane 2–11 successively are: 10^10^, 10^9^, 10^8^, 10^7^, 10^6^, 10^5^, 10^4^, 10^3^, 10^2^, and 10^1^ copies/μL.

Fold dilutions of recombinant plasmids pMD19-T-NS5B were used as templates in the assay using the RAA nucleic acid dipstick assay, which was also compared with the qPCR assay, RAA agarose gel electrophoresis assay, and the conventional PCR assay (Figures 9A–D). The results showed that the minimum detection limit of the CSFV RAA-Strip assay for recombinant plasmid copy number was 10^3^ copies/µL, which was consistent with the sensitivity of the qPCR method, it was 10 fold more sensitive than the CSFV RAA agarose gel electrophoresis method and the CSFV conventional PCR method, indicating that the CSFV RAA-strip method was better sensitive.

**FIGURE 10:**
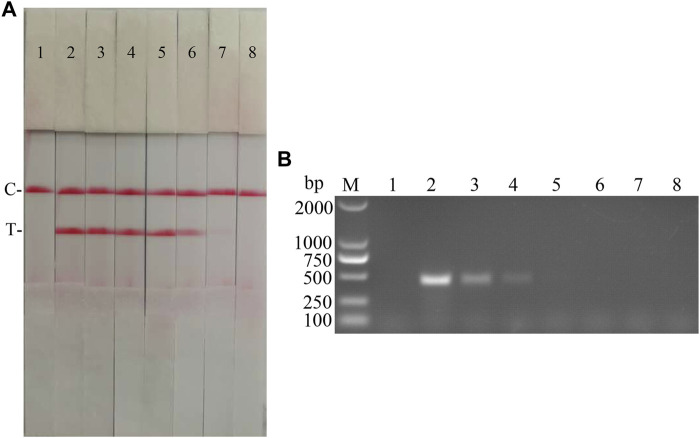
cDNA sensitivity analysis of the CSFV RAA-strip assay. **(A)** The sensitivity test result of CSFV cDNA by CSFV RAA-strip in order. **(B)** was that by the conventional PCR assay. C is a control line. T is a test line. M is a 2,000 bp DNA marker. The templates used in lane 1–8 successively are: negative control, 1.2 × 10^2^ μg/ml, 1.2 × 10^1^ μg/ml, 1.2 μg/ml, 1.2 × 10^−1^ μg/ml, 1.2 × 10^−2^ μg/ml, 1.2 × 10^−3^ μg/ml, 1.2 × 10^−4^ μg/ml.

The sensitivity of the CSFV RAA-strip assay to cDNA was further investigated. The results (Figure 10) showed that the CSFV RAA-strip assay could detect cDNA at a minimum concentration of 1.2 × 10^−2^ μg/ml, that is, the lowest detection limit of cDNA content was 12 pg, which was 100 fold more sensitive than the conventional PCR method for CSFV, indicating that the CSFV RAA-strip method was more sensitive.

**FIGURE 11:**
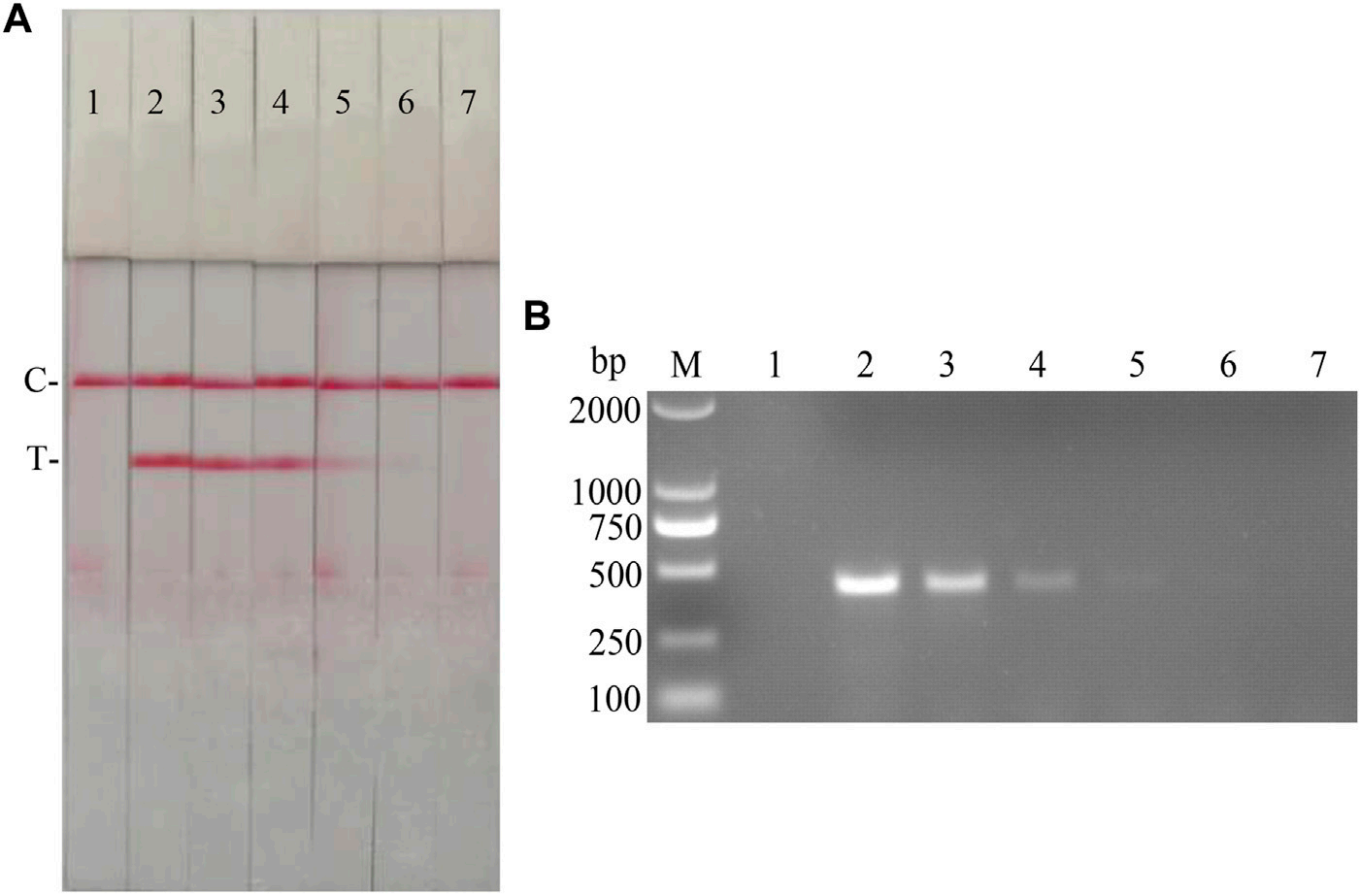
Viral titer sensitivity analysis of the CSFV RAA-strip assay. **(A)** The sensitivity test result of CSFV cDNA by CSFV RAA-strip in order. **(B)** was that by the conventional PCR assay. C is a control line. T is a test line. M is a 2,000 bp DNA marker. The templates used in lane 1–7 successively are: negative control, 10^4.7^, 10^3.7^, 10^2.7^, 10^1.7^, 10^0.7^, and 10^−0.3^ TCID_50_/mL.

The fold diluted CSFV Shimen strain was subjected to nucleic acid extraction and reverse transcription to obtain CSFV cDNA, which was used as the reaction template for detection with CSFV RAA-strips and conventional PCR method, respectively. The results (Figure 11) showed that the CSFV RAA-strip assay could detect 10^1.7^ TCID_50_/mL, which was consistent with the sensitivity of the conventional PCR assay.

#### Clinical Samples Detection

**FIGURE 12:**
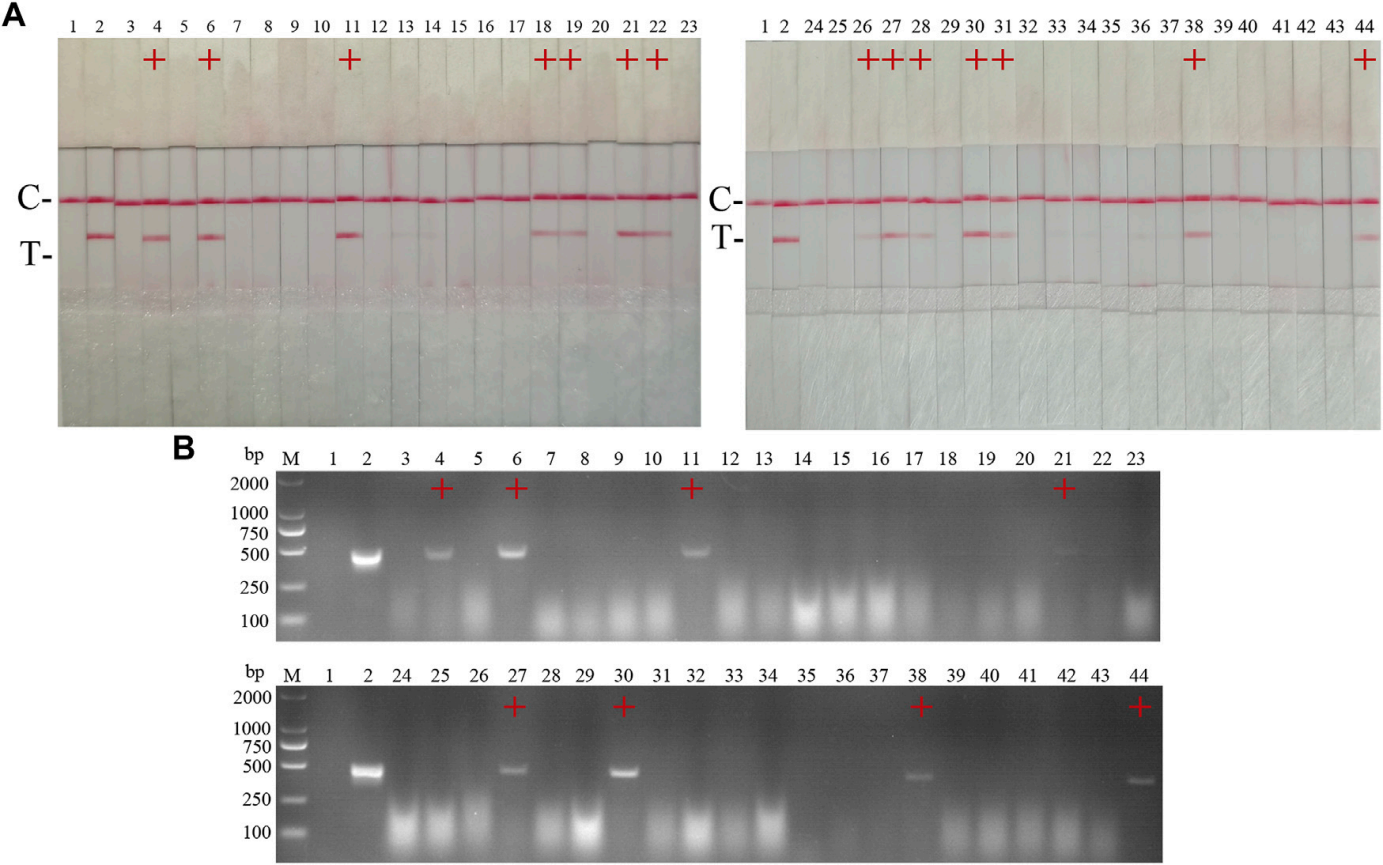
Performance of CSFV RAA-strip on clinical samples. **(A)** The result of clinical samples detection by CSFV RAA-strip. **(B)** The result of clinical samples detection by CSFV conventional PCR. C is a control line. T is a test line. M is a 2,000 bp DNA marker, 1 is a negative control. 2 is positive control (3–44) are different clinical pig samples reserved in our laboratory, and “+” indicates positive detection of CSFV in the samples.

42 clinical samples were examined and comparatively analyzed by the CSFV RAA-strip and conventional PCR, respectively. The results showed that (Figure 12; Table 4), among these samples, 14 positive samples and 28 negative samples were detected by CSFV RAA-strip, with a positive rate of 33.3% and a negative rate of 66.7%; 8 positive samples and 34 negative samples were detected by conventional PCR, giving a positive rate of 19% and a negative rate of 81%. The positive concordance rate was 57.1% between the two methods, while the negative concordance rate was 82.3%, and the overall concordance rate was 85.7%. The results indicated that the CSFV RAA-strip assay had a certain clinical value.

**TABLE 4:** Comparison of CSFV RAA-strip and conventional PCR in clinical samples.

Number of samples	CSFV RAA-strip		Conventional PCR		Coincidence rate
	Positive	Negative	Positive	Negative	
42	14	28	8	34	85.7%

### Development and Preliminary Application of a Dual RAA Agarose Gel Electrophoresis Assay for ASFV and CSFV

#### Establishment of CFSV RAA-Strip and Optimization of the Reaction Condition

**FIGURE 13:**
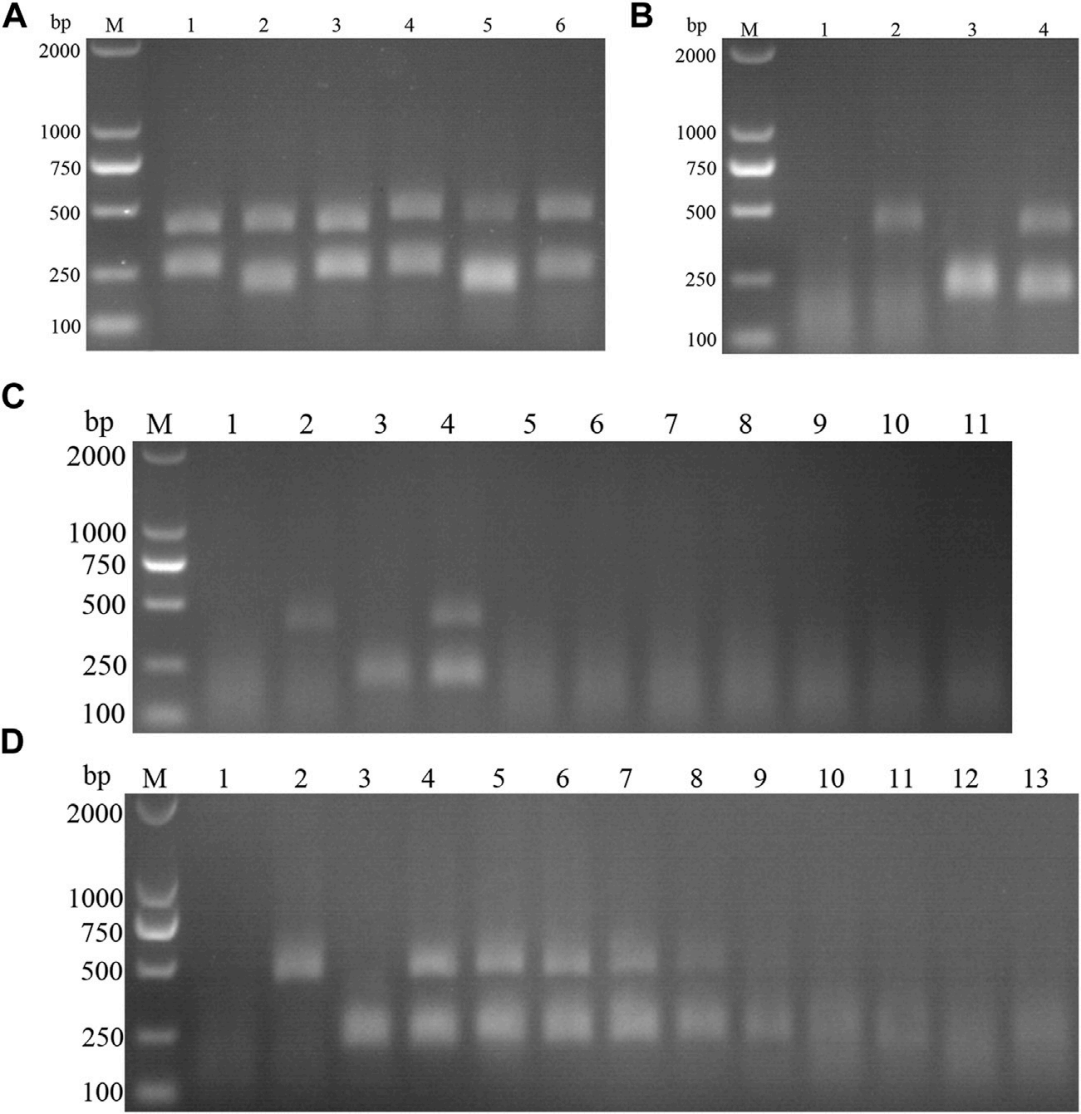
**(A)** Primer screening for dual RAA. M is a 2,000 bp DNA marker. The primers used in lane 1–6 successively are: ASF-RAA-F1/R1 and CSF-RAA F1/R1, ASFV-RAA-F1/R1 and CSF-RAA F2/R2, ASFV-RAA-F1/R1 and CSF-RAA F3/R3, ASFV-RAA-F2/R2 and CSF-RAA F1/R1, ASFV-RAA-F2/R2 and CSF-RAA F2/R2 and ASFV-RAA-F2/R2 and CSF-RAA F3/R3. **(B)** Dual RAA agarose gel electrophoresis of ASFV and CSFV. M is a 2,000 bp DNA marker. The templates used in lane 1–4 successively are the negative control, the ASFV pMD19-T-p72, the CSFV pMD19-T-NS5B, the pMD19-T-p72 + pMD19-T-NS5B. **(C)** Sensitivity of the dual RAA agarose gel electrophoresis of ASFV and CSFV. M is a 2,000 bp DNA marker. Lane 1 is the negative control. The templates used in lane 2–11 successively are ASFV, CSFV, ASFV + CSFV, PPV, PCV2, FMDV, SVV, PRV, PRRSV, and JEV. **(D)** Plasmid sensitivity of the dual RAA agarose gel electrophoresis of ASFV and CSFV. M is a 2,000 bp DNA marker. Lane 1 is the negative control. The templates used in lane 2 and 3 successively are ASFV and CSFV, and the copies of templates used in lane 4–13 successively are 10^10^, 10^9^, 10^8^, 10^7^, 10^6^, 10^5^, 10^4^, 10^3^, 10^2^, and 10^1^ copies/μL.

Specific primers were designed against p72 gene of ASFV, NS5B Gene of CSFV, which resulted in 2 pairs of primers up—and downstream of ASFV, and 3 pairs of primers up and downstream of CSFV (Table 5); Plasmids with the same copy number of ASFV and CSFV, were used as templates to select the best primers for the dual RAA assay. The results (Figure 13A) showed that specific bands corresponding to the expected bands appeared at about 464 and 207 bp for ASF-F1/R1 and CSF-F2/R2, and the brightness of the two bands was not very different and the amplification products were far away, which facilitated the determination of the final detection results, so this primer pair was identified as the best detection primer for the dual RAA agarose gel electrophoresis method of ASFV and CSFV.

**TABLE 5:** Primers of AFSV and CSFV dual RAA used in this study.

Primer name	Sequence (5′-3′)	Product length (bp)
ASF-F1	GGT​TTA​ATG​AGA​ACG​TGA​ACC​TTG​CTA​TTC​CCT​C	464
ASF-R1	GAA​CTT​GTG​CCA​ATC​TCG​GTG​TTG​ATG​AGG	
ASF-F2	CAC​GCT​TGT​AGA​TCC​TTT​TGG​AAG​ACC​CAT	416
ASF-R2	CTT​GTT​TAC​CTG​CTG​TTT​GGA​TAT​TGT​GAG	
CSF-F1	AAA​CGA​CCC​GAG​TTA​GAG​TCC​TCC​TAC​GAT​GCC	260
CSF-R1	TTC​TCA​TCC​ACG​AAG​TCA​CCA​GCG​GTC​CAG​TCA	
CSF-F2	AGA​AGA​AGC​CCA​GAG​TCA​TAC​AAT​ACC​CTG​AA	207
CSF-R2	CCC​ACG​CCT​TAG​TGT​CGA​AGC​TCA​CTG​CTA	
CSF-F3	TGA​TGA​CTG​GAC​CGC​TGG​TGA​CTT​CGT​GGA​T	248
CSF-R3	CCT​GGG​TGT​CCC​ACG​CCT​TAG​TGT​CGA​AGC	

ASFV and CSFV dual RAA agarose gel electrophoresis reactions were performed using ultrapure water as negative control and ASFV and CSFV plasmids as positive templates. The results showed that (Figure 13B), while no bands were detected in the negative control, specific bands and no heterozygous bands appeared in ASFV, CSFV, ASFV, and CSFV mixed templates, indicating that the dual RAA agarose gel method of ASFV and CSFV was developed initially.

#### Specificity and Sensitivity of CSFV RAA-Strip

The DNA templates of PPV, PCV2, FMDV, SVV, PRV, PRRSV, and JEV were used as the specific control group, the mixed templates of ASFV, CSFV and ASFV + CSFV were used as a positive control, and ultrapure water was used as a negative control for RAA amplification. The results showed (Figure 13C) that only ASFV, CSFV, and ASFV + CSFV mixed templates had specific bands consistent with the expected size, and no bands appeared in other templates, indicating the good specificity of the method. Further, using ultrapure water as a negative control, a double RAA agarose gel electrophoresis reaction of double diluted ASFV and CSFV plasmid was carried out. The results showed that (Figure 13D) the minimum detection limit for ASFV P72 gene copy was 10^6^ copies/µL. The minimum detection limit of CSFV NS5B gene copy number was 10^5^ copies/µL.

#### Clinical Samples Detection

**FIGURE 14:**
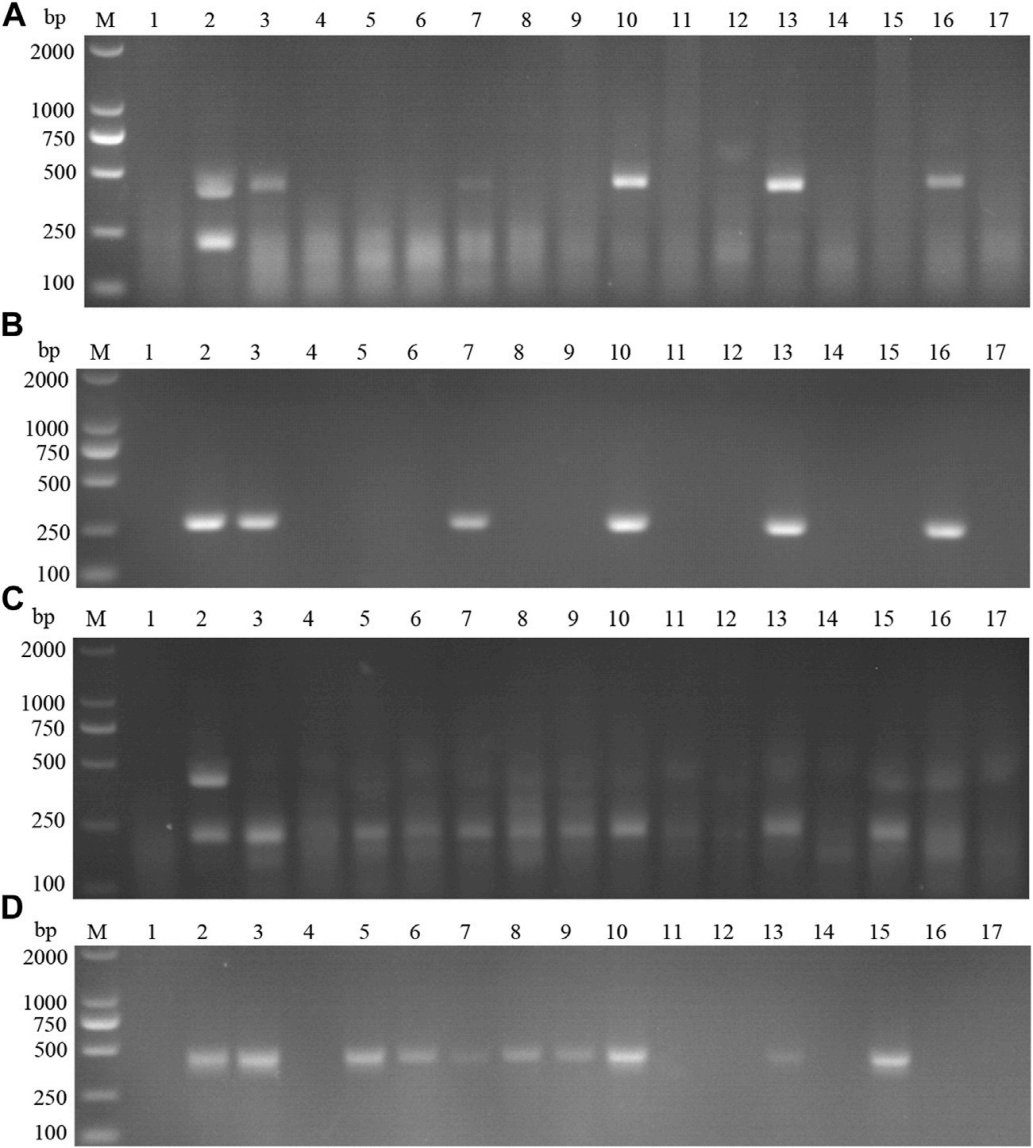
Detection of ASFV suspected clinical materials. **(A)** The result of clinical samples detection by dual RAA agarose gel electrophoresis. M: DL2000 DNA Marker; 1: Negative control; 2: ASFV + CSFV mixed formwork; 3–17: ASFV clinical samples. **(B)** The result of ASFV clinical samples detection by conventional PCR. M: DL2000 DNA Marker; 1: Negative control; 2: ASFV positive template; 3–17: ASFV clinical samples. Detection of CSFV suspected clinical materials. **(C)** The result of clinical samples detection by dual RAA agarose gel electrophoresis. M: DL2000 DNA Marker; 1: Negative control; 2: ASFV + CSFV mixed formwork; 3–17: CSFV clinical samples. **(D)** The result of CSFV clinical samples detection by conventional PCR. M: DL2000 DNA Marker; 1: Negative control; 2: CSFV positive template; 3–17: CSFV clinical samples.

15 suspected infections of CSFV and ASFV were detected by ASFV and CSFV dual RAA agarose gel electrophoresis. The results showed (Table 6) that the dual RAA gel electrophoresis method detected a total of 5 samples of ASF positive disease and 10 samples of negative disease (Figure 14A). Besides, the dual RAA gel electrophoresis was used to detect 9 positive CSF materials and 6 negative cases, all of which were consistent with the results of the conventional PCR method, and the coincidence rate was 100% (Figure 14B). The results showed that the dual RAA gel electrophoresis of ASFV and CSFV has certain clinical practicability.

**TABLE 6:** Comparison of Dual RAA agarose gel electrophoresis and conventional PCR in clinical samples.

Samples	Number of samples	Dual RAA agarose gel electrophoresis		Conventional PCR		Coincidence rate
		Positive	Negative	Positive	Negative	
ASFV clinical samples	15	5	10	5	10	100%
CSFV clinical samples	15	9	6	9	6	100%

## Discussion and Conclusion

ASFV has been spreading since it was first discovered in Shenyang in August 2018, causing huge economic losses to China’s pig industry (Yue et al., 2009). ASF often shares similar clinical symptoms with diseases caused by some other pathogens, for example, CSF, PRRS as well as Salmonella infection, etc. (Oura et al., 2013). Etiological detection methods for ASFV diagnosis mainly include virus isolation and haemadsorption (HAD) test (Moennig and Becher, 2015). Virus isolation can be performed in qualified laboratories with high detection accuracy, but the process of virus isolation is time-consuming and has some requirements for the professional skills of the operators. The HAD can be used for the initial diagnosis of ASFV, and the follow-up needs to be confirmed again by other methods, which is not suitable for rapid on-site detection. The OIE recommended techniques for molecular biology detection of ASFV mainly include common PCR and qPCR methods, however, common PCR detection processes are complicated and prone to cross-contamination, in which qPCR detection, although relatively sensitive, does not have advantages in terms of detection cost, both require sophisticated instrument equipment, and neither is suitable for the analysis of a large number of samples in the first-line of cultivation.

At present, the prevalence of CSF in our country is mainly atypical, usually slowly spreading in swine populations. Serological assays for CSF include viral neutralization test (VNT) and ELISA, among others (Moennig et al., 2003). Among them, VNT is considered the gold standard for CSF antibody detection due to its high sensitivity and specificity. But the method needs to have a certain laboratory infrastructure for work such as cell culture and is prone to cross-contamination and is not suitable for the analysis of large numbers of samples. Etiological detection methods for CSF are mainly virus isolate identification, while this method generally takes a long time and is highly demanding for personnel, suitable for in-depth studies of CSFV such as research institutes or schools, and not suitable for clinical detection. Among molecular biology detection methods qPCR is usually the first choice for CSFV nucleic acid detection, with the advantages of high detection sensitivity and specificity, which is widely used in disease diagnosis and scientific research, but usually requires specialized instrument equipment (Bai et al., 2019). Therefore, it is important to establish a rapid and accurate clinical diagnostic method as the first line of defense to prevent and control CSF epidemics.

RAA, a novel isothermal nucleic acid amplification technology developed based on recombinase polymerase amplification (RPA), has Chinese autonomous intellectual property rights, its recombinase is derived from bacteria or fungi, is more accessible than phage recombinase in RPA technology, the advent of this technology has made the field of isothermal amplification technology more abundant, and has strongly promoted the development of *in vitro* nucleic acid amplification technology. The RAA technology offers several advantages in terms of detection technique and methodology, firstly, the core part of RAA is to mix the three enzymes to make a lyophilized enzyme powder, a move favoring transport and storage and not producing much attrition during use due to repeated freeze-thawing. Second, RAA technology has higher sensitivity compared with traditional PCR technology, and the specificity is also guaranteed, with shorter reaction time and easier operation. The RAA technology fills the gap in traditional culture and technology that relies on thermolabile equipment and is expected to become an alternative to PCR for nucleic acid amplification in the future. Currently, RAA technology has been widely used in viruses (Zhang et al., 2019) bacteria, parasites (37), and so on.

Immunochromatographic nucleic acid detection dipstick technology has many advantages, such as easy operation, intuitive results, fast detection, and rapid color development by adding tiny amounts of RAA amplified products to the dipstick sample pad in a buffer, which has many more outstanding advantages over general conventional methods, such as personnel and equipment requirements. In this study, we combined the RAA nucleic acid amplification technique with immunochromatographic nucleic acid strip assay, which targets the ASFV p72 gene, the CSFV NS5B Gene, and the RAA-strip assay for ASFV or CSFV respectively, and the amplification of the target nucleic acid can be completed within 15 min, and the detection results can be observed in 5 min. The RAA nucleic acid strip assay for ASFV can detect plasmid copy numbers as low as 10^3^ copies/µL. The minimum detection limit for ASFV DNA was 10 pg, with a positive concordance rate of 94.1% compared with conventional PCR in terms of clinical sample detection. The RAA-strip assay for CSFV can detect plasmid copy numbers as low as 10^3^ copies/µL. The minimum detection limit of cDNA for CSFV was 12 pg, and the positive concordance rate between detection of clinical test samples and conventional PCR was 57.1%. The RAA-strip assay is suitable for clinical on-site detection, can effectively and quickly know the detection of the samples to be examined, meets the need of rapid clinical detection in China, and is an ideal and generalizable test. However, this method still has some deficiencies, most importantly sample processing before product detection, and nucleic acid extraction of the pending samples is time-consuming when the number of samples is large. Second, there are strict requirements for the design of primers and probes, which will generate a certain cost during the primer design screening process. In addition, nucleic acid test strips, although they have the advantage of rapid detection, also have the problem of not being quantitative, which will create some limitations to the wide application of this method.

Agarose gel electrophoresis is commonly used to identify and purify DNA fragments, which has the advantages of easy operation and low cost. In this study, we developed a dual RAA gel electrophoresis assay for ASFV and CSFV by combining the RAA technique with agarose gel electrophoresis. The dual RAA gel electrophoresis assay, similar to the dual PCR method, is capable of identifying two different pathogens simultaneously, however the multiplex PCR method has strict requirements for a companion reaction instrument, and the reaction process is limited by space, time, cost, etc., which greatly hinders the promotion of on-site detection of this technique. LAMP has complex primer design procedures, takes more than an hour to complete nucleic acid amplification, and is not suitable for testing large batches of samples. The dual RAA gel electrophoresis of ASFV and CSFV established in this study does not need expensive instrument equipment, has convenient operation and less time-consuming reaction, which meets the demand of rapid detection of mixed infection of clinical pathogens, is suitable for remote or poorly equipped areas, and has a promising application in disease prevention and control. The key point of this technology is the design and selection of primers, and when designing the primers, care should be taken to avoid the formation of primer-dimers and hairpin structures, and the possibility of hetero bonds if the target fragment binds nonspecifically to the sequence, which is not conducive to the determination of the final detection results. In addition, care needs to be taken to make a suitable choice for the size of the fragment of interest for the amplification primers, and the observation that the amplified fragments differ by a larger size is more favorable for results. The dual RAA agarose gel electrophoresis assay for ASFV and CSFV established in this study showed good specificity and a high positive detection rate, which provided a new idea for the differential diagnosis of isothermal amplification products. However, the present method also has some defects, and after the RAA amplification reaction is completed, the amplified products need to be purified using a chloroform solution, and this step increases the operation time to some extent.

In summary, this study established an RAA-strip assay for ASFV/CSFV and a dual RAA agarose gel electrophoresis assay for ASFV and CSFV from a time-saving and labor-saving point of view, and the above methods have the advantages of easy operation and high detection efficiency, which may provide a new idea for clinical rapid detection and differential diagnosis of ASFV and CSFV. With the continuous progress and development of science and technology, the direction and research hotspot of RAA technology will be more around quantitative detection and multiple pathogen co-detection. If different biological labels are added to the test strips, it is possible to achieve simultaneous and rapid detection of different pathogens, which is of great importance in the rapid diagnosis of mixed infection diseases and is expected to be more popularized in areas with poor environmental resources and some sudden outbreaks.

## Data Availability

The original contributions presented in the study are included in the article/Supplementary Material, further inquiries can be directed to the corresponding authors.
