# Supplementary material for: Development and Application of RAA Nucleic Acid Test Strip Assay and Double RAA Gel Electrophoresis Detection Methods for ASFV and CSFV
Source: Front Mol Biosci. 2022 Jan 31;8:811824. doi: 10.3389/fmolb.2021.811824 (PMC8841470; doi:10.3389/fmolb.2021.811824)
Supplement: Supplementary file 1 [file Table1.docx]

Supplementary Material

**Supplementary Table 1.** Primers of AFSV/CSFV conventional PCR used in this study.

| **Primer name** | **Sequence (5’-3’)** | **Position (nt)** |
| --- | --- | --- |
| OIE-ASFV-F | CTTACCGATGAAAATGATAC | 772-791 |
| OIE-ASFV-R | ATGGATACTGAGGGAATAGC | 1049-1030 |
| OIE-CSFV-F | GACACAAGCGCAGGCAATAG | 1583-1602 |
| OIE-CSFV-R | AGTGGGTTCCAGGACTACAT | 2031-2012 |
