# Supplementary material for: Development and Application of RAA Nucleic Acid Test Strip Assay and Double RAA Gel Electrophoresis Detection Methods for ASFV and CSFV
Source: Front Mol Biosci. 2022 Jan 31;8:811824. doi: 10.3389/fmolb.2021.811824 (PMC8841470; doi:10.3389/fmolb.2021.811824)
Supplement: Supplementary file 2 [file Table2.docx]

Supplementary Material

**Supplementary Table 2.** Primers of AFSV/CSFV qPCR used in this study.

| **Primer name** | **Sequence (5’-3’)** | **Position (nt)** |
| --- | --- | --- |
| qASF-F | CAGGAGGTATCGGTGGAGGGAAC | 688-710 |
| qASF-R | GGTTTGCTTTGGTGCGGCTTG | 767-747 |
| qCSF-F | CCTGAGGACCAAACACATGTTG | 685-706 |
| qCSF-R | TGGTGGAAGTTGGTTGTGTCTG | 858-837 |
