# Supplementary material for: Development and Application of RAA Nucleic Acid Test Strip Assay and Double RAA Gel Electrophoresis Detection Methods for ASFV and CSFV
Source: Front Mol Biosci. 2022 Jan 31;8:811824. doi: 10.3389/fmolb.2021.811824 (PMC8841470; doi:10.3389/fmolb.2021.811824)
Supplement: Supplementary file 3 [file Table3.docx]

Supplementary Material

**Supplementary Table 3.** Primers of AFSV recombinase aided amplification (RAA) used in this study.

| **Primer name** | **Sequence (5’-3’)** | **Product length (bp)** |
| --- | --- | --- |
| ASF-RAA-F1 | AGAATGCGTACCGAAACTTGGTTTACTACTGGCT | 288 |
| ASF-RAA-R1 | GCGTATCATTTTCATCGGTAAGAATAGGTT |  |
| ASF-RAA-F2 | CACGCTTGTAGATCCTTTTGGAAGACCCAT | 416 |
| ASF-RAA-R2 | CTTGTTTACCTGCTGTTTGGATATTGTGAG |  |
| ASF-RAA-F3 | CAAGCCGCACCAAAGCAAACCTATTCTTACCG | 282 |
| ASF-RAA-R3 | AGGTTCACGTTCTCATTAAACCAAAAGCGCAAC |  |
